# Oral supplementation with capsaicin reduces oxidative stress and IL-33 on a food allergy murine model

**DOI:** 10.1016/j.waojou.2019.100045

**Published:** 2019-07-02

**Authors:** Maísa Mota Antunes, Bruna Scherr Laignier Coelho, Thaís Makiya Vichi, Elandia Aparecida dos Santos, Fabíola Karine Braga Gondim, Ariane Barros Diniz, Edenil Costa Aguilar, Denise Carmona Cara, Laura Cristina Jardim Porto, Isabela Coelho de Castro, Jacqueline I. Alvarez Leite, Lílian Gonçalves Teixeira

**Affiliations:** a Center for Gastrointestinal Biology, Departamento de Morfologia, Universidade Federal de Minas Gerais, Belo Horizonte, Minas Gerais, 31270-901, Brazil; b Departamento de Bioquímica e Imunologia, Instituto de Ciências Biológicas, Universidade Federal de Minas Gerais, Belo Horizonte, Minas Gerais, 31270-901, Brazil; c Departamento de Nutrição, Universidade Federal de Lavras, mailbox: 3037, Lavras, Minas Gerais, 37200-000, Brazil

**Keywords:** Allergy, Capsicum, Intestinal mucosa

## Abstract

**Background:**

Food allergy is an abnormal immune response to antigens introduced into the body through food. Its prevalence has increased in developed and developing countries. Natural products are traditionally used to alleviate and treat diseases, and diet can play a role in both the prevention and management of food allergy. The effects of capsaicin as an anti-oxidant, anticarcinogenic, and anti-inflammatory in the energy expenditure and suppression of fat accumulation have been demonstrated. This study evaluated the effect of oral supplementation with capsaicin on a food allergy model.

**Methods:**

OVA-sensitized mice received ovalbumin solution, and they were fed with chow supplemented with capsaicin for 7 days. The control group received AIN-93 chow with no supplementation. IgE anti-ova, inflammatory infiltration, oxidative stress and metabolic analysis were performed.

**Results:**

The results showed that capsaicin supplementation is not able to reduce characteristic signs of food allergy, such as production of IgE and weight loss. However, macrophages infiltration and IL-33 in proximal jejunum was reduced in OVA capsaicin group. In addition, hepatic triglycerides and intestinal hydroperoxides were reduced in both capsaicin groups.

**Conclusion:**

Oral supplementation with capsaicin attenuated important factors associated to food allergy such as inflammation and oxidative stress, suggesting better prognosis and evolution of the disease.

## Background

Food allergy is the manifestation of an abnormal immune response to antigens introduced into the body through food. A group of disorders is triggered after consumption of certain foods in individuals with sensitivity to these antigens.^1^ In these individuals, allergy is related to the presence of antibodies specific for food antigens in the serum and is frequently associated with high serum immunoglobulin E (IgE) levels.^2^ Its prevalence has increased in developed and developing countries, and it affects between 1.1 and 10.4% of children.^3^, ^4^ Diet can play a role in both the prevention and management of food allergy.^5^

The use of natural products, especially those derived from medicinal plants, is a traditionally used way to alleviate the disease and was described from remote times in different civilizations. Capsaicinoids are the compounds responsible for the spicy taste of pepper, and the primary capsaicinoid of pepper is capsaicin.^6^, ^7^ The effects of capsaicin such as anti-oxidant,^8^ anticarcinogenic,^9^ and anti-inflammatory^10^ in energy expenditure and suppression of fat accumulation^11^ have been demonstrated.

Investigations of the effect of capsaicin on food allergy have not yet been performed. Considering the changes in lipid metabolism due to food allergy,^12^ and the anti-inflammatory effect of capsaicin,^10^, ^13^ as well as its protective effect on gastric mucosa,^14^ in this study it was hypothesized that capsaicin could improve some inflammatory and oxidative parameters in food allergy, and it could act on the liver, contributing to the control of lipid metabolism.

## Material and methods

### Animals and experimental design

This study was approved according to the protocol CEUA/UFMG 127/2014. Male BALB/c mice, 6- to 8-weeks old, were obtained from “Biotério Central” in Universidade Federal de Minas Gerais (UFMG, Brazil) and conditioned under controlled conditions of temperature (24 ​°C) and luminosity (light/dark cycle of 12/12 ​h), with chow (AIN-93)^15^ and water *ad libitum*. The animals were randomized into 4 groups: Non-allergic ovalbumin (OVA) negative (OVA-) control group (no food allergy and no capsaicin supplementation), Allergic (OVA+) group (with food allergy and no capsaicin supplementation), Non-allergic capsaicin control group (no food allergy and 0.01% capsaicin supplemented in the chow), and Allergic ​+ ​Capsaicin group (with food allergy and 0.01% capsaicin supplemented on chow). The body weight was measured once per week during the 28 days of experimental challenge, and chow and water intake were measured every day throughout the experiment.

On day 28, the animals underwent euthanasia, and the blood, liver, and small intestines were harvested for the assessment of intestinal histopathology, oxidative stress levels, and enzyme and biochemical assays.

All animal studies were in accordance with the Animal Care and Use Committee at UFMG, and were carried out according to the standards set forth in the Guide for the Care and Use of Laboratory Animals of the National Research Council.

### OVA sensitization and oral challenge

The OVA-sensitized mice (OVA ​+ ​group) received subcutaneous (SC) injection of 0.2 ​mL saline with 1 ​mg Al(OH)_3_, as adjuvant, and 10 ​μg OVA (five times crystallized hen's egg albumin; Sigma Chemical Co., USA) on day 0 and saline with 10 ​μg OVA on day 14. Mice from Control group (OVA−) received a SC injection of 0.2 ​mL saline with adjuvant on day 0 and saline on day 14. One week after the administration of the booster, the oral challenge was conducted (day 21). For all groups, drinking water was replaced with a 20% OVA solution for 1 week and quantified each day to verify aversion. This solution was prepared using a lyophilized egg white (Salto's, Belo Horizonte, Brazil).^16^, ^17^ At the same time point, chow (AIN-93) of capsaicin groups was supplemented with 0.01% of capsaicin.^18^, ^19^, ^20^, ^21^ The other groups received AIN-93 chow with no supplementation.

### Confirmation of allergic response – serum IgE

Anti-OVA IgE ELISA was performed using plates coated with rat anti-mouse IgE (Southern Biotechnology Associates, Birmingham, USA), serum, and biotinylated OVA, as previously described.^22^ The reaction was developed with the streptavidin-peroxidase conjugate (Extravidin; Sigma), plus o-phenylene-diamine (OPD) and H_2_O_2_. The plate was read at 492 ​nm and the results were reported in arbitrary units (AU) using a positive reference serum as being 1000 units.

### Cytokines and enzyme assays

Samples (50 ​mg) of proximal jejunum were homogenized and centrifuged. Supernatants were used for cytokine levels quantification, and precipitates were used for quantification of enzyme activities. The levels of Interleukin (IL)-33, Tumor Necrosis Factor (TNF) and IL-10 from small intestine were quantified using DuoSet ELISA kits according to the manufacturer's instructions (R&D Systems, Inc., Minneapolis, MN, USA). Neutrophil, Macrophage and Eosinophil infiltrations were evaluated by analyzing the enzyme activity of myeloperoxidase (MPO), N-acetylglucosaminidase (NAG) and eosinophil peroxidase (EPO), respectively, as previously described.^23^, ^24^, ^25^

### Total liver lipids and metabolic analysis

For hepatic lipid measurements, 100 ​mg of liver tissue was homogenized at 4°C in lysis buffer containing 50 ​mM Tris (pH 8.0), 150 ​mM NaCl and 1% Triton X-100. Lipids were extracted from the total liver homogenate using the chloroform-and-methanol method.^26^ Cholesterol, and triacylglycerol levels from liver and serum, and fasting glucose in the serum were assayed using enzymatic kits according to the manufacturer's instructions (Katal, Belo Horizonte, Brazil).

### Oxidative stress

Samples of the small intestine were washed with phosphate-buffered saline (PBS) to remove the intestinal contents, and fragments from liver were washed. Evaluation of lipid peroxidation by thiobarbituric acid reactive substances (TBARS) and the hydroperoxide concentrations were performed as previously described.^24^

### Histopathologic analysis

Histopathologic analysis was performed as previously described.^24^ Briefly, the small intestine was removed, measured, gently perfused with paraformaldehyde (3%), divided into the duodenum, jejunum and ileum and fixed in paraformaldehyde (3%) for 24 ​h. The segments were dehydrated, embedded in paraffin, and cut into 5-μm-thick sections before being stained with hematoxylin and eosin (H&E). Digital images were obtained using a 10x objective on a light microscope (Olympus, Center Valley, PA) fitted with a digital camera (Moticam 500, Motic, British Columbia, Canada) for histopathologic evaluation. Alterations of the mucosal architecture (general structure, cell distribution, mucosal and submucosal aspect), the presence of ulcerations, villus height, and the extent of inflammatory infiltration were used to determine the histologic score. The samples were coded and then scored by a trained pathologist who was not aware of the treatment modalities.

### Statistical analysis

Experimental data were tested for determination of outliers (Grubbs’ test) and normality using the Kolmogorov–Smirnov test. One-way ANOVA and TUKEY multiple comparisons post-test were used in the analysis of different groups. Student t-test was used for comparison between correlated groups. Statistical analysis was performed using the Graph Pad Prism 5.0^®^ software package (San Diego, California). All data are given as the mean ​± ​s. e.m (standard error of the mean). Experimental groups had at least five mice per group. Data shown are representative of at least two independent experiments. Differences were considered as significant at *p* ​< ​.05.

## Results

### OVA challenge induces allergy in mice, but capsaicin supplementation is not able to reduce characteristic signs of allergy

**Fig. 1:**
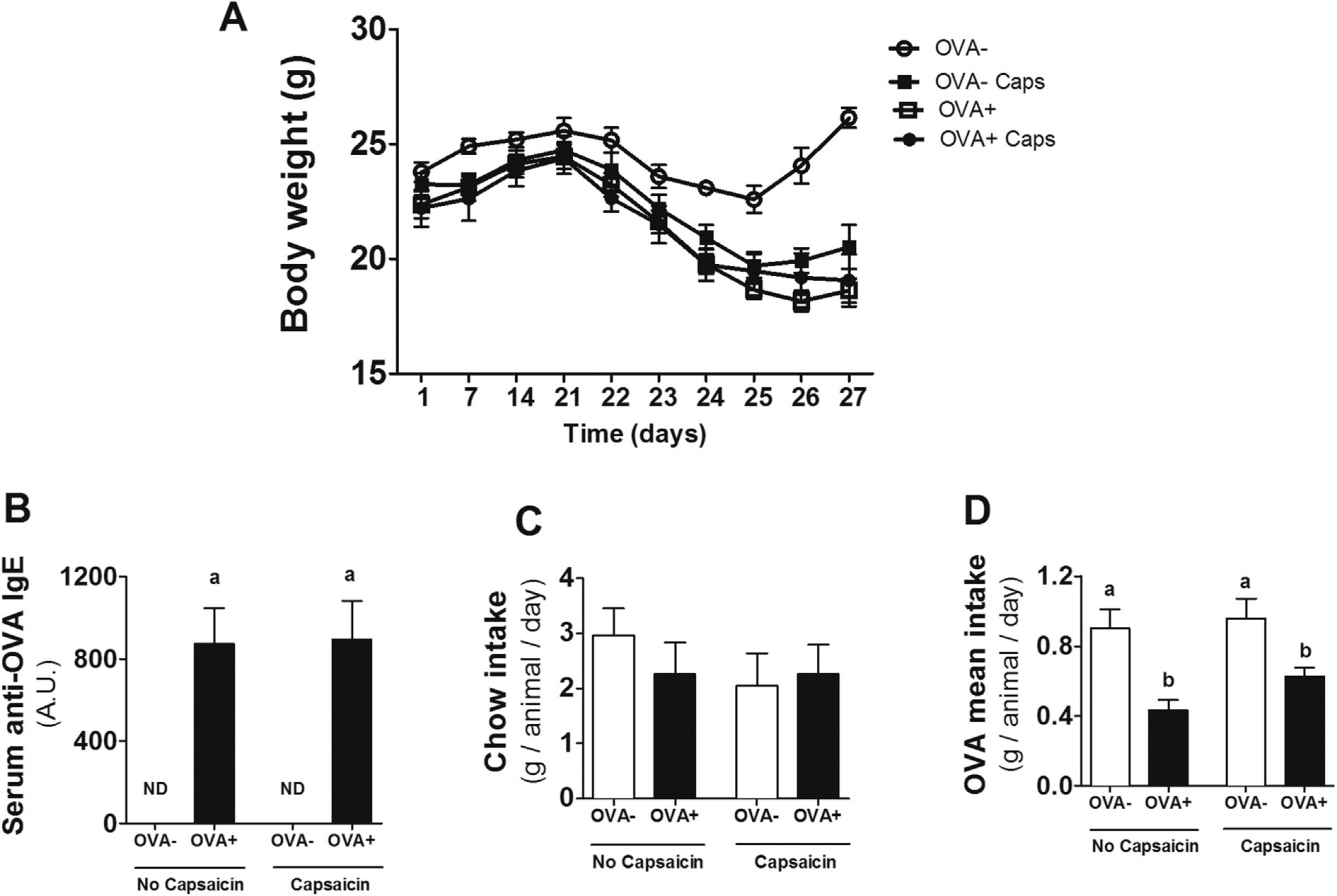
**Confirmation and clinical evaluation of food allergy. (A)** Body weight throughout the experimental design. **(B)** Marker of allergy, serum levels of anti-OVA IgE in nonsensitized (OVA−) and sensitized (OVA+) mice after oral ovalbumin challenge. Evaluation of **(C)** Chow intake and **(D)** OVA consumption. Results expressed as mean and standard error. Different letters represent significant differences. ANOVA One-way analysis of variance and Newman-Keuls multiple-comparison post test were used (*p* ​< ​.05). Number of mice in each group ≥10.

Loss of body weight and increased levels of IgE are characteristic signs of food allergy.^17^ Here, the lower weight gain and the higher levels of this immunoglobulin attested to the development of allergy to the OVA protein (Fig. 1A and B). The similarity in food consumption between groups confirms that the lower weight gain is due to the development of food allergy. Sensitized mice (OVA+) also demonstrated aversion to the antigen in water. In fact, allergic groups had lower consumption of water containing OVA, which was not observed on non-allergic mice (Fig. 1D). Although there has been success in the development of OVA-induced allergic responses, capsaicin supplementation was not able to reverse these parameters.

### Metabolic changes

**Fig. 2:**
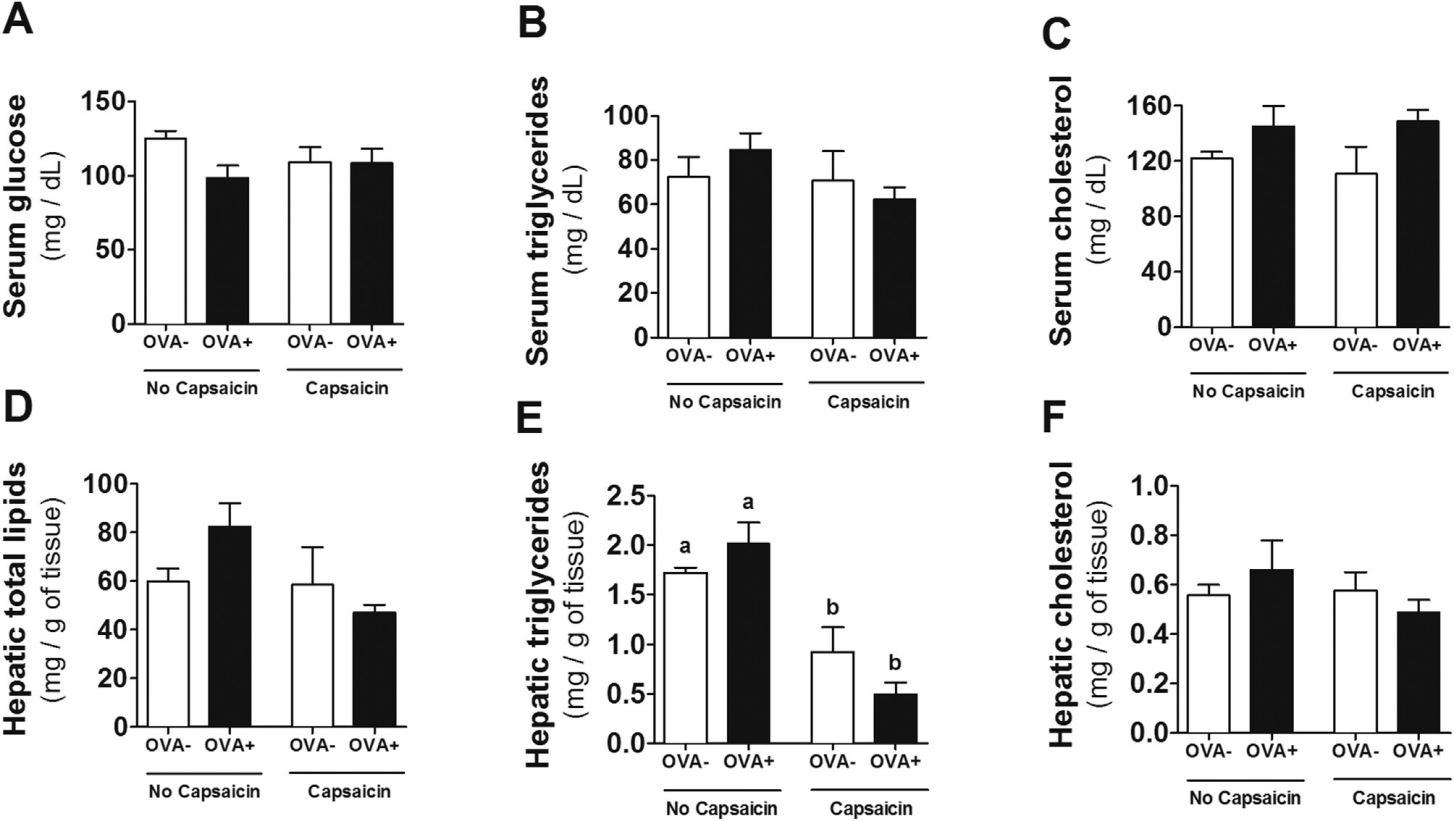
**Biochemical analysis of liver and serum during OVA-mediated food allergy.** Metabolic changes induced by OVA challenge and capsaicin supplementation were evaluated by serum levels of **(A)** Glucose, **(B**) Triglycerides, and **(C)** Cholesterol, and hepatic levels of **(D)** Total lipids, **(E)** Triglycerides, and **(F)** Cholesterol. Results expressed as mean and standard error. Different letters represent significant differences. ANOVA One-way analysis of variance and Newman-Keuls multiple-comparison post test were used (*p* ​< ​.05). Number of mice in each group ≥5.

The metabolic effects of capsaicin on insulin resistance, hepatic steatosis, and lipid and obesity reduction have been demonstrated.^18^, ^19^, ^21^, ^27^ However, no changes were observed in serum glucose, total cholesterol and triglycerides (Fig. 2A–C) in our model. In the evaluation of hepatic metabolism, although no changes were observed between the groups for total lipids and cholesterol (Fig. 2D and F), capsaicin was able to reduce triglycerides in both groups with and without food allergy (Fig. 2E).

### Histological analyses

**Fig. 3:**
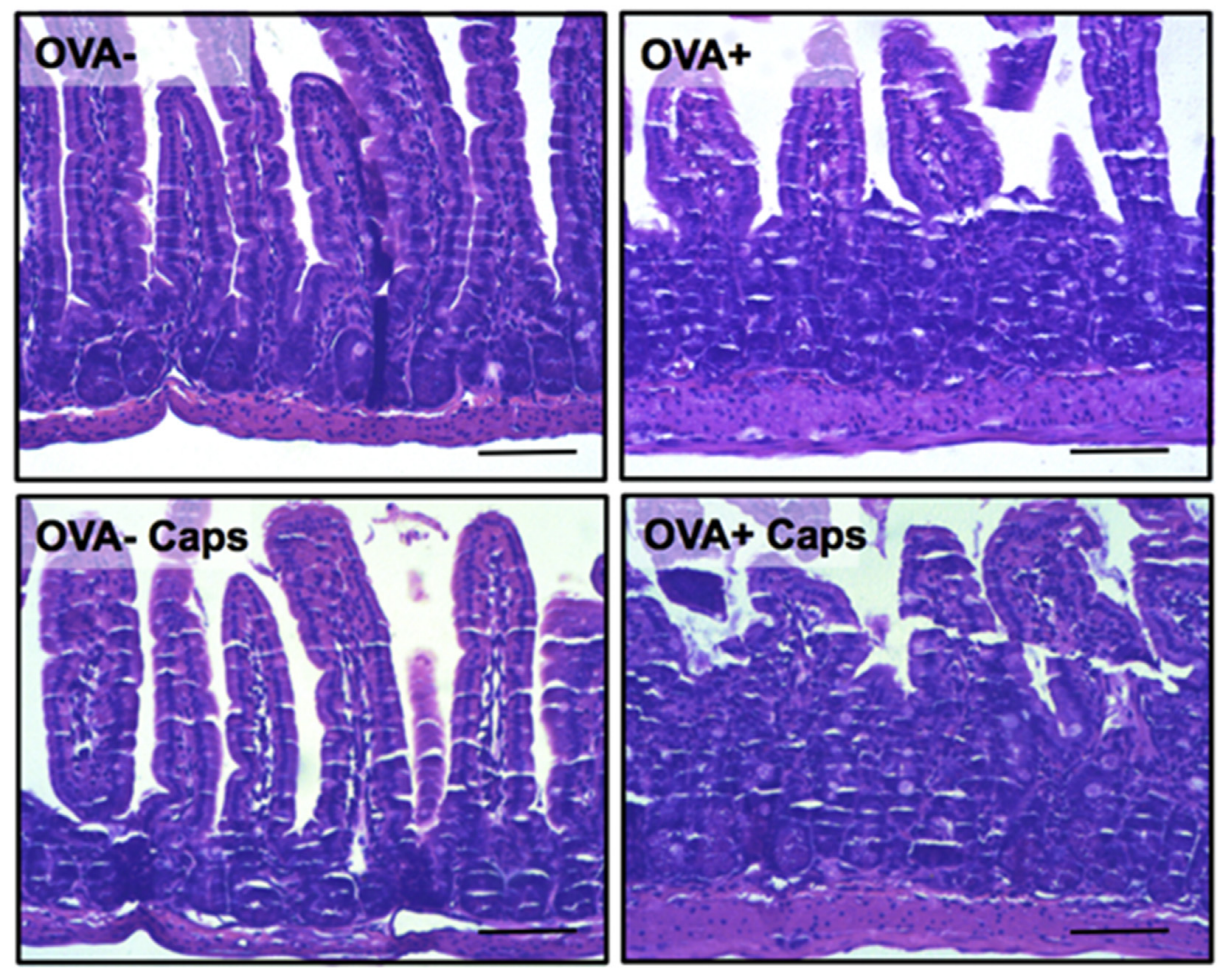
**Morphologic structure of the proximal jejunum does not change with capsaicin supplementation.** Morphology of the proximal jejunum after OVA challenge. 10x magnification. Hematoxilin & Eosin. Images representative of at least 5 animals per group. Scale ​= ​100 ​μm.

The proximal jejunum is the intestinal portion (small intestine) most affected by food allergy.^17^ The histopathological evaluation during the food allergy indicates that the proximal jejunum of the allergic animals presented tissue damage in relation to the control animals, with decreased villus height and loss of mucosal architecture. However, capsacin supplementation was not able to minimize the damage caused by food allergy (Fig. 3). Furthermore, the histopathological evaluation of allergic animals showed that both capsaicin-treated and untreated groups did not confirm the presence of inflammatory infiltrate in the tissue.

### Enzyme activity assay

**Fig. 4:**
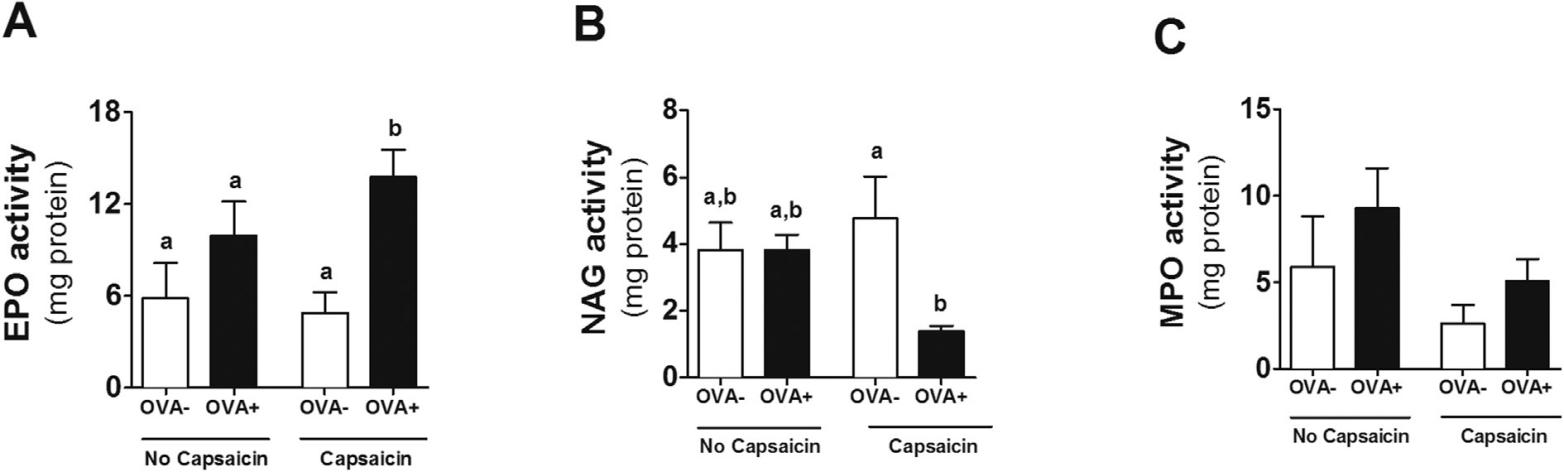
**Eosinophil and macrophages are altered due to capsaicin supplementation.** Assessment of **(A)** EPO, **(B)** NAG and **(C)** MPO activity in the proximal jejunum. Results expressed as mean and standard error. Different letters represent significant differences. ANOVA One-way analysis of variance and Newman-Keuls multiple-comparison post test were used (*p* ​< ​.05). Number of mice in each group ≥5.

In the histopathological evaluation, no evidence of change in the number of inflammatory cells was observed in the allergic animals or in the capsaicin-treated animals. However, although the number of inflammatory cells is not changed, the activity of these cells may be different. We then decided to investigate the possible relevance of inflammatory infiltrate by the evaluation of specific enzymes present in each cell type. By indirect evaluation of eosinophil peroxidase activity, we observed that the combination of food allergy with capsacin led to increased eosinophils in the proximal jejunum (Fig. 4A). However, we observed that the supplementation of capsaicin in allergic animals led to a decrease in the activity of NAG (indirect measurement of macrophages) in relation to its control in the proximal jejunum (Fig. 4B), in addition to a decrease in neutrophil myeloperoxidase activity among groups, although this decrease is not significant (Fig. 4C).

### Cytokines assay

**Fig. 5:**
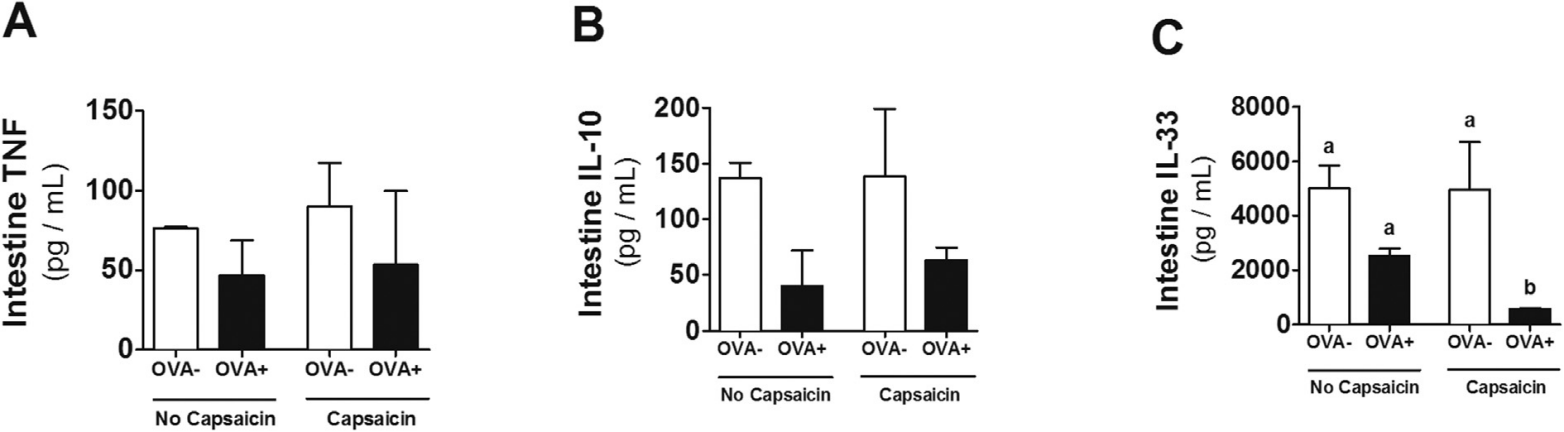
**Cytokines analysis of proximal jejunum during OVA-mediated food allergy.** Levels of **(A)** TNF, **(B)** IL-10 and **(C)** IL-33 were estimated by ELISA. Results expressed as mean and standard error. Different letters represent significant differences. ANOVA One-way analysis of variance and Newman-Keuls multiple-comparison post test were used (*p* ​< ​.05). Number of mice in each group ​= ​5.

The presence of pro-and anti-inflammatory cytokines is closely related to the inflammation as well as the resolution process. In view of this, and considering previous results, the next step was to investigate the concentrations of some cytokines in the proximal jejunum. TNF concentrations did not change in any group (Fig. 5A), regardless of allergy or capsaicin supplementation. The allergic and non-allergic capsaicin-treated groups showed a trend of reduction in IL-10 concentrations (Fig. 5B). Interestingly, the capsaicin-supplemented allergic group had a reduction in IL-33 levels in the proximal jejunum (Fig. 5C).

### Oxidative stress

**Fig. 6:**
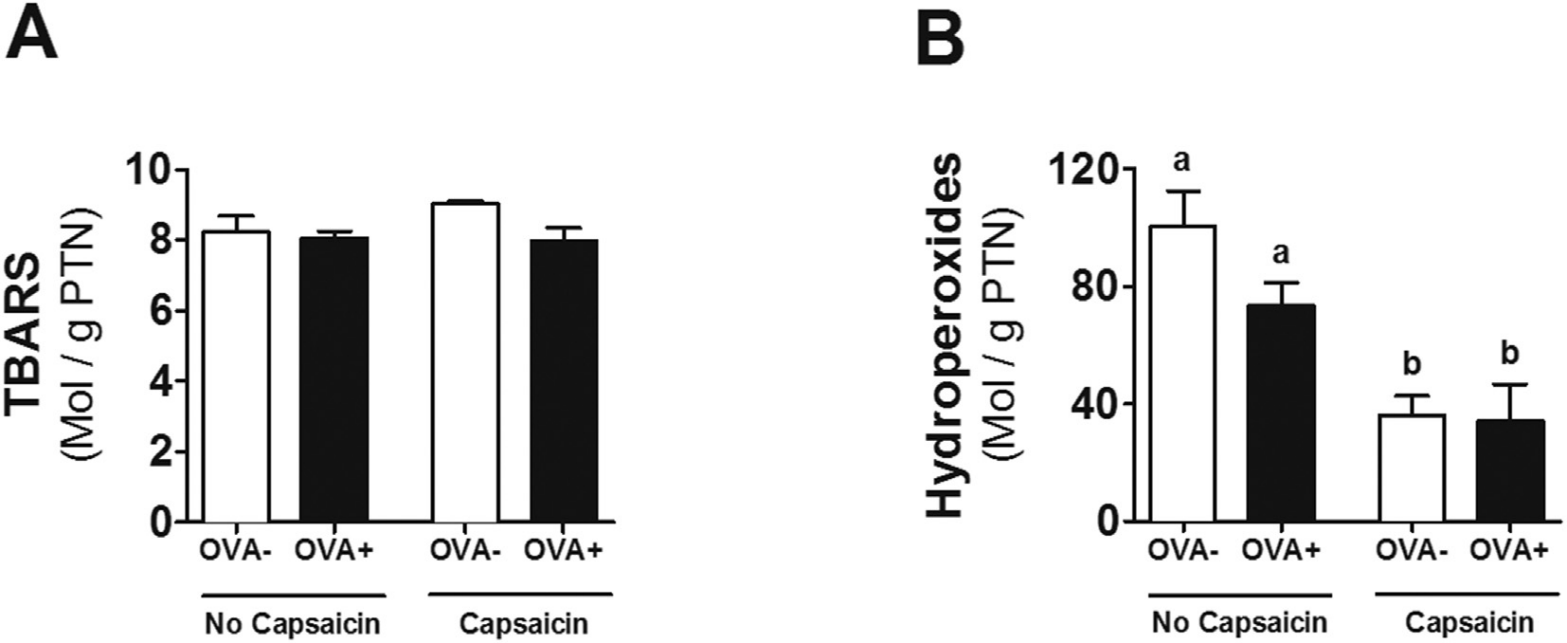
**Capsaicin supplementation decreases oxidative stress in proximal jejunum**. Analysis of **(A)** lipid peroxidation by thiobarbituric acid reactive substances (TBARS) and **(B)** concentration of hydroperoxide. Results expressed as mean and standard error. Different letters represent significant differences. ANOVA One-way analysis of variance and Newman-Keuls multiple-comparison post test were used (*p* ​< ​.05). Number of mice in each group ≥5.

Considering that no differences were found in cytokine concentrations that could explain the inflammatory profile in allergy, the next step was to investigate the effect of capsaicin supplementation on oxidative stress, since the antioxidant action of capsaicin has already been described.^8^ We observed that, although the lipid peroxidation by TBARS (Fig. 6A) did not change between the groups, the reduction in hydroperoxide concentrations confirms the anti-oxidant action of capsaicin (Fig. 6B).

## Discussion

Previous studies have linked the use of capsaicin with attenuation of oxidation, inflammation and fat accumulation in a large spectrum of diseases.^8^, ^10^, ^11^ However, the involvement of capsaicin in the pathogenesis of food allergy — widely distributed in developing countries - was unknown. In this way, the objective of this work was to evaluate the effect of capsaicin in a food allergy murine model. Here we showed that oral supplementation with capsaicin attenuates important signs of allergy such as allergy inflammation and oxidative stress which suggests possible benefits in the food allergy. To the best of our knowledge, this is the first work to evaluate this effect.

Food allergies have been known to cause weight loss and increase IgE levels.^17^ In food allergy models in which ovalbumin is offered in the diet, food aversion is observed in the animals, with consequent lower food intake and weight loss.^17^ Because of this, in this study, ovalbumin was offered in water to avoid interfering with the intake of capsaicin that was supplemented in the diet. We observed a similar food intake between all groups (Fig. 1C), and no food aversion was observed in the animals. We also observed that mice receiving ovalbumin had lower weight gain and increased IgE levels (Fig. 1A and B). However, capsaicin was not able to alter these parameters related to food allergy.

Interestingly, lower weight gain was observed in control mice that consumed capsaicin compared to untreated control animals (Fig. 2A), and this was not associated with lower chow intake, since the average food consumption was similar between groups (Fig. 2C). Topical action of capsaicin as a thermogenic compound has been demonstrated, leading to less weight gain or weight loss in healthy individuals.^13^ The allergic treated-mice presented final body weight similar to the non-allergic treated-mice, suggesting that there was no synergistic or additive effect between allergy and capsaicin in inducing less weight gain. This result may suggest that in situations with higher energy demand, such as in the high inflammation of active phase of food allergy,^28^, ^29^ the action of capsaicin as thermogenic is annulled.

The reduction of hepatic triglycerides induced by capsaicin comsuption has been demonstrated in obese mice.^27^, ^30^ In inflammatory diseases, adipose tissue lipolysis occurs, with lipid release for use as a substrate for immune cells.^12^, ^29^ Thus, the action of capsaicin on the regulation of lipid metabolism is important in order to avoid abnormal fat deposition if these lipids were not used as an energetic substrate.

Although the anti-inflammatory and anti-oxidant actions of capsaicin have been described,^8^, ^10^ in this study these actions were not determinant in the local damage caused by food allergy in the intestine (Fig. 3). This may be explained by a shift of these macrophages to another inflammatory site or by a preference for other inflammatory cells, as seen with eosinophils. In addition, the reduction of macrophage infiltration into adipose tissue has already been reported as an effect of capsaicin,^30^ enhancing its anti-inflammatory action. The increase of eosinophils is expected in the model of food allergy used. These cells are responsible for the late phase of the immediate allergic reaction.^17^ Eosinophils are mucosal cells, such as the gastrointestinal and respiratory tract. Increased eosinophils are associated with increased production of inflammatory cytokines such as IL-1, IL-6 and TNF.^31^ Thus, increased eosinophils observed in the allergic group that consumed capsaicin (Fig. 4A) may be one of the factors by which capsaicin was not able to improve food allergy parameters topically, even though oxidative stress in the same tissue was reduced (Fig. 6B).

Despite elevated levels of EPO, we found a significant decrease in the levels of IL-33 (Fig. 5C). IL-33 has been considered as an emerging key factor in the development of allergic diseases, and its reduction can improve then by reducing pro-inflammatory cytokine production and Th2 type immune cell activation.^32^ Endogenous IL-33 enhances Th2 cytokine production during allergic airway inflammation, contributing to peripheral antigen-specific responses in ovalbumin-induced acute allergic lung inflammation.^33^ Interestingly, the presence of IL-33 can deregulate Treg cells in the lung leading to changes in airway tolerance.^34^ Additionally, prevention of food allergy development and suppression of established food allergy by neutralization of IL-33 has been shown.^35^ These findings suggest an important effect of capsaicin in our model.

The antioxidant action of capsaicin has already been described.^8^ However, to our knowledge, this is the first work in which the anti-oxidant action of capsaicin (Fig. 6B) is described in food allergy. Interestingly, oxidative stress has been shown to be associated with increased expression of IL-33.^36^, ^37^ Thus, the reduced production of IL-33 in OVA capsaicin animals may be the result of lower oxidative stress in this group. Although these effects were not sufficient for an improvement in the mucosal architecture of allergic animals, a reduction of free radicals through consumption of an anti-oxidant food may bring better prognosis and evolution of the disease.^38^

## Conclusion

Oral supplementation with capsaicin was able to attenuate important factors associated to allergy such as inflammation and oxidative stress, resulting in lower IL-33 production in murine model of food allergy. These effects suggest possible benefits in the prognosis and evolution of the disease. Perhaps the intake for longer times and constant consumption of capsaicin may be able to alleviate food allergy. More studies should be performed for evaluation.

## Abbreviations

AIN-93: standard chow
AU: arbitrary units
CEUA: ethics committee of animal experimentation
EPO: eosinophil peroxidase
H&E: hematoxylin and eosin
Ig: Immunoglobulin
IL: Interleukin
MPO: myeloperoxidase
NAG: N-acetylglucosaminidase
OPD: o-phenylene-diamine
OVA: Ovalbumin
PBS: phosphate-buffered saline
TBARS: thiobarbituric acid reactive substances
TNF: Tumor Necrosis Factor
UFMG: Universidade Federal de Minas Gerais

## Declarations

### Availability of data and materials

“Please contact author for data requests.”

## Authors' information

Our group investigates intestinal diseases, with inflammatory profile, among them, food allergy, and the role of several nutrients as adjuvants in the prevention and treatment of these. Capsaicin has been studied in our group in several situations in which it could act as an antioxidant and anti-inflammatory. LGT, JIAL and DCC are leaders of groups that develop these studies, being DCC specialist specifically in food allergy.
